# Nitrogen supply influences photosynthesis establishment along the sugarcane leaf

**DOI:** 10.1038/s41598-018-20653-1

**Published:** 2018-02-02

**Authors:** Denis Bassi, Marcelo Menossi, Lucia Mattiello

**Affiliations:** Departamento de Genética, Evolução, Microbiologia e Imunologia, Instituto de Biologia, Universidade Estadual de Campinas, 13083-862 Campinas, Brazil

## Abstract

Nitrogen (N) is a major component of the photosynthetic apparatus and is widely used as a fertilizer in crops. However, to the best of our knowledge, the dynamic of photosynthesis establishment due to differential N supply in the bioenergy crop sugarcane has not been reported to date. To address this question, we evaluated physiological and metabolic alterations along the sugarcane leaf in two contrasting genotypes, responsive (R) and nonresponsive (NR), grown under high- and low-N conditions. We found that the N supply and the responsiveness of the genotype determined the degree of senescence, the carboxylation process mediated by phosphoenolpyruvate carboxylase (PEPcase) and differential accumulation of soluble sugars. The metabolite profiles indicated that the NR genotype had a higher respiration rate in the youngest tissues after exposure to high N. We observed elevated levels of metabolites related to photosynthesis in almost all leaf segments from the R genotype under high-N conditions, suggesting that N supply and the ability to respond to N influenced photosynthesis. Therefore, we observed that N influence on photosynthesis and other pathways is dependent on the genotype and the leaf region.

## Introduction

Sugarcane (*Saccharum spp* L.) is considered to be a renewable feedstock for economically valuable products, such as sugar and bioethanol^1,2^. Moreover, the biomass remaining after sugar and bioethanol production can be burned to generate bioelectricity or used for production of second-generation ethanol (2G-bioethanol)^3,4^. The capacity of sugarcane to produce high amounts of biomass and accumulate high a sucrose content (approximately 700 mM) in the mature stalks^5^ is the result of a combination of various factors, including an efficient C4 photosynthesis system that allows high yields of dry matter per hectare^6^.

Photosynthesis is influenced by many environmental factors, such as atmospheric CO_2_ concentration, light incidence, temperature and water availability^7–11^. Furthermore, the specific leaf nitrogen (SLN) content positively affects photosynthesis^12^, which is partly related to nitrogen (N) partitioning in photosynthetic enzymes, pigment content and the size, number and composition of chloroplasts^13–16^. Meinzer & Zhu^17^ observed that photosynthesis increases linearly with the increase in leaf N content in sugarcane. The remobilization of N from leaves to stalks during the reproductive stage causes a reduction in photosynthesis^18,19^. More recently, biochemical induction of photosynthesis in response to changing irradiance was faster under high-N conditions in rice^20^. N also stimulated leaf growth through the synthesis of proteins involved in cell growth, cell division, and cell wall and cytoskeleton synthesis^21^, increasing the photosynthetic area. In maize, an increase of 29% in leaf area was observed under high N supply compared to that under low N supply^22^. Although many studies have reported a positive influence of N on photosynthesis, only one report in wheat^23^ has shown how N influences photosynthesis establishment and cell differentiation along the leaf blade.

Leaf senescence is also influenced by N and is related to a decline in photosynthetic capacity. The photosynthetic proteins of plastids are extensively degraded in an early phase of senescence compared to other proteins^24,25^. For both annual and perennial plants, this process contributes to N remobilization from senescent leaves to growing organs and seeds^26,27^. Despite the dearth of studies on leaf senescence process in sugarcane, one study^28^ observed that N, P, and K had higher levels of remobilization than did B, Cu, Fe and Zn, between different leaves and in different regions of the same leaf (base, middle and tip). In addition, N remobilization was more pronounced in the tip and middle regions, where senescence is more visible. Conversely, in wheat, an abundant N supply delays leaf senescence, which results in maintenance of photosynthesis^29^. In species with a longer growing period such as sugarcane, this delay in leaf senescence may contribute to sugar accumulation in the leaves and subsequent sucrose accumulation in the stalks during maturation.

The identification and quantification of metabolites is a powerful tool for plant biology studies. Metabolites are the ultimate products derived from biological reactions and are therefore useful phenotypic markers, providing a better understanding of specific pathways related to agronomically important traits and their changes due to stress conditions^30^. Despite the complexity of studies of metabolites in plants, such studies have expanded in the past several years, but data from sugarcane are scarce^31^. Considering that grass leaves have a well-defined developmental gradient with a cell division zone at the base followed by cell elongation and maturation zones^32–34^, the use of metabolic profiling can be helpful for understanding the C4 photosynthesis establishment process and leaf development. Pick *et al*.^32^ observed that some metabolites accumulated to different levels along the maize leaf blade. C4 malate, C4 acids, TCA cycle intermediates and C4 pyruvate clusters were elevated towards the leaf base, whereas the tip contained elevated amounts of drought-related metabolites in the raffinose family. Another study compared the metabolic profile of different segments of maize and rice leaves and observed that C3 and C4 species have specific metabolite signatures concerning amino acids levels and other organic acids involved in photosynthesis^35^. Furthermore, Stitt^36^ proposed that large pools of metabolites such as malate, aspartate (Asp), alanine, triose phosphates and glycerate-3-phosphate have the potential for buffering ATP and NADP, providing enormous flexibility and robustness in relation to light fluctuations. Regarding sugarcane, there have been relatively few studies using metabolomics to assess sucrose accumulation^31^, stem development^30^ and genotypic characterization^37^.

This study investigated the influence of N on the establishment of photosynthesis and other processes of primary metabolism related to growth and leaf development by analysing changes in physiological parameters and metabolic profiles at different developmental stages of sugarcane leaf using two contrasting genotypes. We discuss the differences in responsiveness of photosynthetic performance to N with the aim of opening new venues to improve carbon fixation and, consequently, biomass production in sugarcane.

## Materials and Methods

### Plant material and experimental conditions

The selection of the two contrasting genotypes (RB975375 – responsive (R); RB937570 – nonresponsive (NR)) was based on a screening of N-use efficiency (NUE) according to Robinson^38^ with 20 genotypes and three N concentrations (10, 90 and 270 mg of N per kg of sand; Supplementary Fig. S1). NUE analysis was based on biomass production in relation to N uptake. Culms provided by the Inter-University Network for the Development of Sugarcane Industry (Ridesa, Brazil) were germinated inside a greenhouse in trays containing vermiculite. Three weeks after germination, plantlets were transferred to plastic pots filled with 3.4 kg of washed sand and kept in a greenhouse with a constant temperature of approximately 28 °C. The application of macro- and micronutrients (Supplementary Table S1) was based on previous soil chemical analyses. Ammonium nitrate was used as the N source and applied in two different concentrations (10 and 270 mg of N per kg of sand). N was applied three times at an interval of 15 days. The experimental design was completely randomized with three repetitions.

### Collection of leaf segments

**Figure 1:**
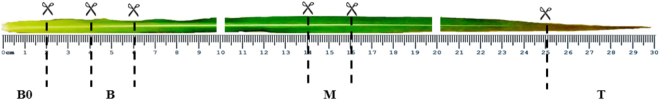
Schematic of segments of sugarcane leaf + 1 that were collected. B0, Base Zero; B, Base; M, Middle; and T, Tip.

One month after the last N application (three-month-old plants – see Supplementary Fig. S2), different segments of leaf +1 (first completely expanded leaf that had a visible dewlap and was photosynthetically active) were collected according to Mattiello *et al*.^39^. First, the total length of the leaf was measured and divided into three along the leaf blade (Fig. 1). The first two centimetres of each leaf were called “Base Zero” (B0). The middle regions of the first two thirds were called “Base” (B) and “Middle” (M), and the end of final third was called “Tip” (T). The segments from leaves of the same age were collected between 10 am and 2 pm and frozen in liquid N.

### Photosynthetic parameters

Photosynthesis rate (A), stomatal conductance (gs) and transpiration rate (E) were measured in the M of leaf +1 using a portable infrared gas exchange meter (LI-6400-40, LiCor, USA). The CO_2_ value was fixed at 400 μmol.mol^−1^, light intensity at 1,500 μmol.m^2^.s^−1^, and leaf temperature at 28 ± 1 °C. Photosynthetic NUE (PNUE) was calculated as described by Marchiori *et al*.^13^, Hirel *et al*.^40^ and Pons and Westbeek^41^.

### **Measurements of** leaf **N and carbon isotopic discrimination**

The total N, carbon content and carbon isotopic discrimination were quantified at the UC Davis Stable Isotope Facility (USA) using one plant per replicate. Approximately 3–4 mg of dried and ground material from each segment was placed in tin capsules and sent for analysis. Samples were analysed using Elemental PDZ Europa ANCA-GSL interfaced with a mass spectrometer PDZ Europa with an isotopic rate of 20–20 (Sercon Ltd., UK). Results were provided as δ13 (stable carbon isotopes rate, represented by negative values and in ‰). To determine the magnitude of isotopic fractionation between plant and atmosphere, we calculated the Δ13C (carbon isotopic discrimination, represented by positive values) according to Farquhar^42^.

### Quantification of chlorophylls

Chlorophyll *a* and *b* contents were determined in ethanol solution as described by Cross^43^, using one plant per replicate. Absorbance readings were measured in a microplate spectrophotometer (Infinite 200 PRO NanoQuant, Tecan, CH), and readings were taken at wavelengths of 665 and 645 nm for chlorophyll *a* and *b*, respectively.

### **Enzyme** activity **and quantification of PEPcase and RubisCO**

To assess the *in vitro* activity of the enzymes, 50 mg FW was mixed with extraction buffer as described previously by Gibon^44^, using two plants per replicate. Both enzymes were measured spectrophotometrically at a wavelength of 340 nm. The reaction mixture, containing enzyme extract, 25 mM Tris-HCl pH 8, 5 mM MgCl_2_, 4 mM DTT, 5 mM NaHCO_3_, 5 mM glucose-6-phosphate, 5 mM PEP, 0.2 mM NADH and 2 U MDH, was used for phosphoenolpyruvate carboxylase (PEPcase) assays. To assay Ribulose-1,5-bisphosphate carboxylase/oxygenase (RubisCO) activity, the reaction mixture was prepared according to methods described by Lilley^45^ and contained enzyme extract, 100 mM bicine/NaOH pH 8, 20 mM MgCl_2_, 25 mM NaHCO, 3.5 mM phosphocreatine, 3.5 mM ATP, 0.25 mM NADH, 4.8 U of G3PDH, 4.8 U of creatine phosphofructokinase, 4.8 U of G3P kinase and 0.5 mM RuBP. PEPcase and RubisCO levels were estimated by western blot using enzyme-specific antibodies.

### **Metabolic** profiling **analysis**

Leaf segments comprising three biological replicates from a pool of two plants were ground to a fine powder, and 50 mg of fresh material was mixed with 1 mL of a precooled (−15 °C) mixture of MTBE:methanol:water 3:1:1 (v/v/v), as described previously by Giavalisco^46^. The organic phase was dried and derivatized according to Roessner^23^. Derivatized samples were analysed on a Combi-PAL autosampler (Agilent Technologies, USA) coupled to an Agilent 7890 gas chromatograph and a Leco Pegasus 2 time-of-flight mass spectrometer (LECO, St. Joseph, MI, USA). Chromatograms were exported from Leco ChromaTOF software (version 3.25) to R. Peak detection, retention time alignment, and library matching were performed using the Target Search R-package^47^. The intensity of each metabolite was normalized by dividing each value by the dry weight of the sample and by the sum of the total ion counts. To further correct for measurement effects, values were then normalized by the median of all the measured data and log2 transformed. To determine sample structure and the presence of distinct groups within the dataset, partial least square discriminant analysis (PLS-DA) was performed using mixOmics R package^48^. To determine which metabolic pathways were enriched based on all metabolites identified in each sample, a metabolite set enrichment analysis (MSEA)^49^ with an over-representation analysis (ORA)^50^ approach was performed using the MetaboAnalyst^49^ software (version 3.0). A customized metabolite set library containing 39 pathways was created using KEGG Pathway^51^ and PlantCyc in Plant Metabolomic Network (PMN)^52^ as metabolic pathway reference database. The fold enrichment was calculated based on the expected random number of metabolites in each pathway and the observed number at a 5% significance level and 10% false discovery rate (FDR). Hierarchical clustering analysis coupled to a heatmap was generated using R software. The significance of the levels of metabolites was also tested by unpaired t-test. Two analysis strategies were applied (see Supplementary Fig. S3).

### **Statistical** analysis

Data were submitted to variance analysis using the R package Agricolae – version 1.2 to verify the interactions among and within factors (genotype, treatment and leaf segments). Fisher’s Least Significant Difference^53^ was applied at a 5% significance level.

### Data availability

The datasets generated during and/or analysed during the current study are available from the corresponding author on reasonable request

## Results and Discussion

### **Response of** sugarcane **genotypes to differential N supply**

Plants exhibit a remarkable ability to sense environmental factors, such as N supply, and it is common to observe an array of genetic variation leading to different responses even in the same species. We observed that the mean A, gs and E values in the R genotype (RB975375) were nearly twice as high as those in the NR genotype (RB937570) in the 270 N treatment (270 mg of N per kg of sand; see Supplementary Fig. S4). Furthermore, the genotypes showed contrasting profiles in some biometric traits (see Supplementary Information) evaluated under high-N conditions (Supplementary Fig. S5). According to principal component analysis (PCA) of 17 traits, in the 10 N treatment (10 mg of N per kg of sand), the two genotypes were grouped into a single cluster (Supplementary Fig. S6), indicating that both had a similar response to low-N conditions. On the other hand, in the 270 N treatment, two distinct clusters were formed (Supplementary Fig. S6), indicating a contrasting response of the two genotypes. The NR genotype may present changes in physiological and biochemical traits, but not to the extent observed in the R genotype.

### **Chlorophyll** content **increases in response to N towards the leaf tip**

**Figure 2:**
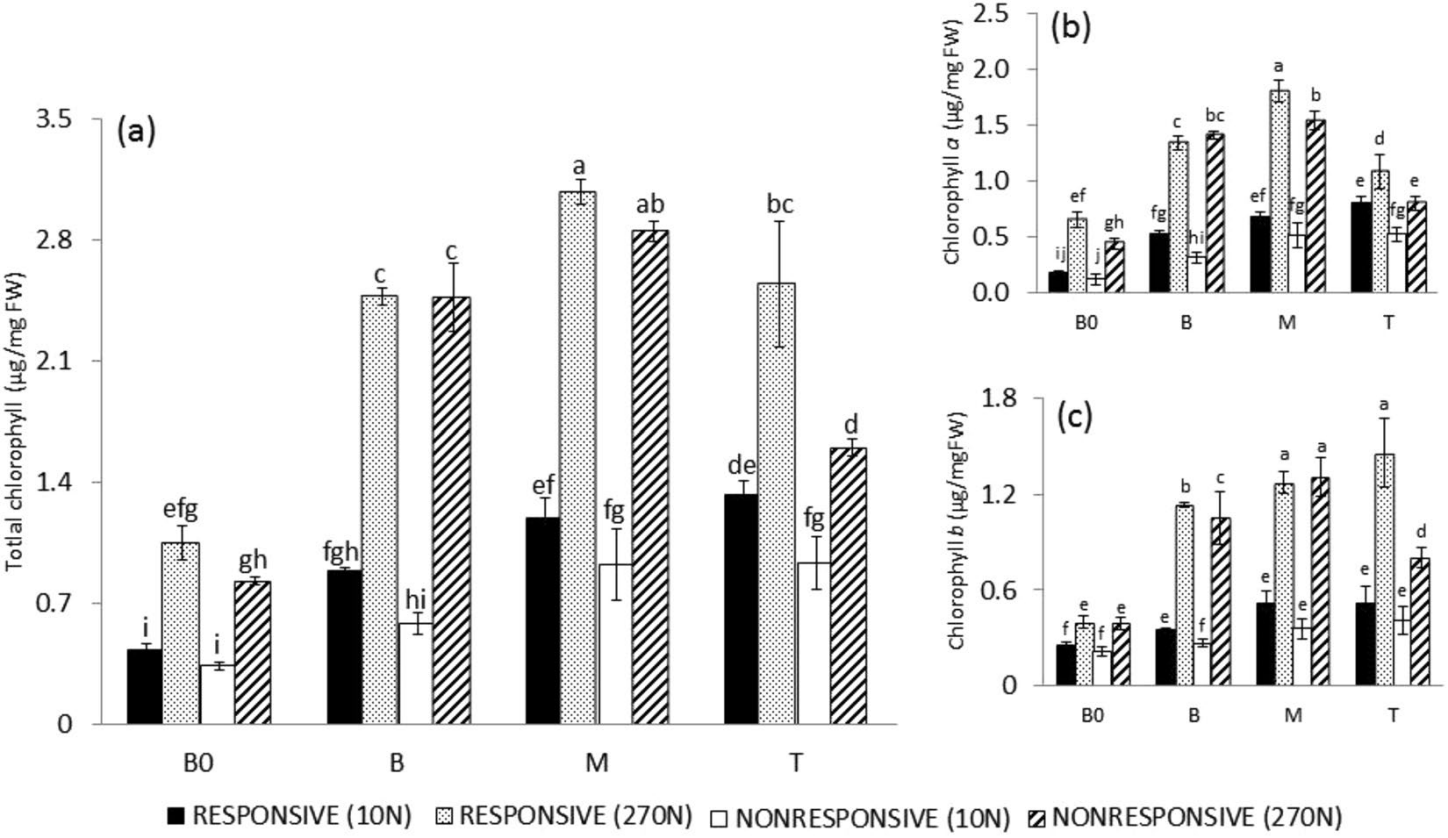
Chlorophyll content in different segments of sugarcane leaf blade. (**a**) Total chlorophyll; (**b**) chlorophyll *a*; (**c**) chlorophyll *b*. 10 N and 270 N correspond to treatments of 10 and 270 mg of N per kg of sand, respectively. B0, Base “zero”; B, Base; M, Middle; T, Tip. FW, fresh weight. Data are presented as the mean ± SE. Letters indicate statistical significance using ANOVA followed by Fisher’s exact test (n = 3; P ≤ 0.05).

Quantification of chlorophylls is considered an important parameter to verify the concentration of pigments involved in light absorption and energy transfer during the photochemical process of photosynthesis. Decreases in chlorophyll content may mark the start of senescence. Furthermore, the chlorophyll molecule contains N, making this element an important factor in the development of the photosynthetic apparatus in plants. There is evidence that C_4_ plants invest approximately 30% of leaf N in thylakoid components such as chlorophylls^54^. The total chlorophyll content increased under high-N supply in all segments (B0, B, M and T) along the leaf blade in both genotypes (Fig. 2a), consistent with other studies that showed an increase in leaf chlorophyll content in sugarcane plants cultivated under high-N supply in different environments^55,56^. In addition, total chlorophyll concentration considerably increased from the B0 to M segments in 270 N, ranging from 1.05 to 3.07 µg/mg FW in the R genotype and from 0.83 to 2.84 µg/mg FW in the NR genotype, as shown in Fig. 2a. Distinct chlorophyll concentrations along leaf segments indicate a photosynthetic gradient in sugarcane, in the same fashion as observed previously in studies of other sugarcane cultivars and in other grass species, such as maize and wheat^23,32,33,39^.

In contrast with the B0 segment, which presented the lowest total chlorophyll content under high-N supply, the M and B segments had the highest amounts of total chlorophyll in the 270 N treatment in both genotypes (Fig. 2a), indicating that N investment in chlorophyll is more extensive in this portion of the leaf, where cells are at a more mature developmental stage^32^, primarily in relation to plastid development. With the exception of the B region, all other segments presented a higher chlorophyll *a* content in the 270 N treatment in the R genotype than in the NR genotype (Fig. 2b). The B and T levels of chlorophyll *b* were higher in the R genotype (Fig. 2c). In addition, despite the difference in chlorophyll *b* levels in the B region between the R and NR genotypes under high-N conditions being small, statistical analysis showed that the difference was significant.

Differences in the total chlorophyll and chlorophyll *a* and *b* contents between the two genotypes under high-N conditions were clearer in the T segment (Fig. 2), with higher levels in the R genotype. In grass leaves, such as those of sugarcane, the start of senescence, characterized by degradation of proteins and plastids, is more evident in the M and T regions of leaves compared to regions next to the basal region^28^. In addition, transcriptional data from different segments along the sugarcane leaf blade revealed that some senescence-associated genes were up regulated in the T region^39^. Our data indicate a delay in leaf senescence symptoms of R plants cultivated under high-N supply, which could be due to a better balance between chlorophyll degradation and synthesis in these plants.

### **Carboxylation process** responds **better to N supply in the middle leaf region**

**Figure 3:**
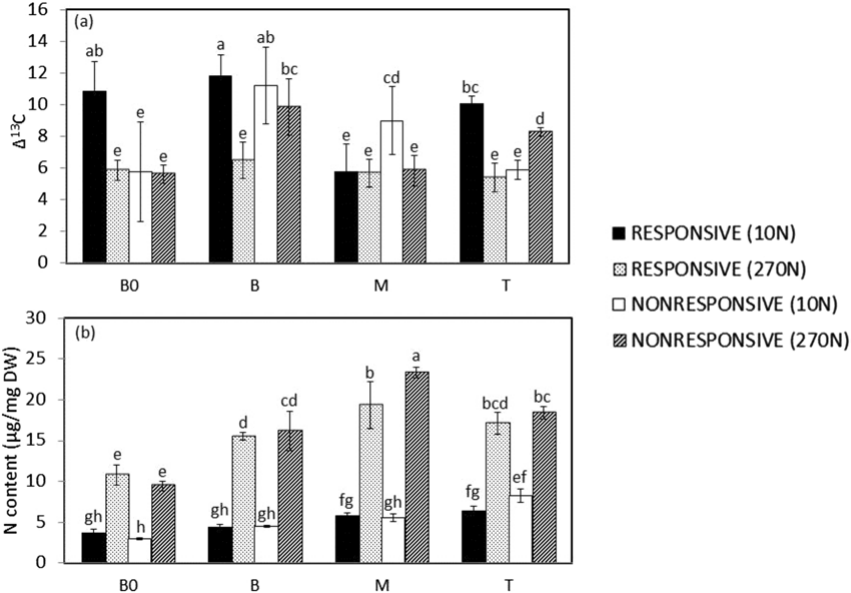
(**a**) Carbon isotopic discrimination (Δ^13^C) and (**b**) total leaf N content along sugarcane leaf blade. B0, Base “zero”; B, Base; M, Middle; T, Tip. 10 N and 270 N correspond to treatments of 10 and 270 mg of N per kg of sand, respectively. DW, dry weight. Data are presented as the mean ± SE. Letters indicate statistical significance using ANOVA followed by Fisher’s exact test (n = 3; P ≤ 0.05).

Carbon isotope (Δ^13^C) discrimination is used to characterize C_4_ photosynthetic responses in plants grown under diverse environments and stresses^57–59^. A greater decrease in Δ^13^C was observed in all segments in the R genotype under high-N supply (Fig. 3a), indicating that the carboxylation process mediated by PEPcase responded more to the N supply in this genotype than in the NR genotype, mainly because PEPcase has lower discrimination for ^13^C than does RubisCO^60^. The reduced Δ^13^C measured in sugarcane leaves in response to 270 N could also be related to the rate of CO_2_ leakiness^57,58^. However, leakiness rates (see Supplementary Information) did not significantly differ and ranged from 0.055 ± 0.003 to 0.054 ± 0.005 at the M leaf region in the R and NR genotypes in the 270 N treatment, respectively. For T segment, a significant increase in Δ^13^C in the NR genotype compared to the R genotype was observed (Fig. 3a). This result might be related to the intensity of the senescence process in the NR genotype, as evidenced by the decrease in chlorophyll content in the leaf tip (Fig. 2).

Observations indicate that in C_4_ plants, the total N allocation in RubisCO and PEPcase ranged from 5–9% and 2–5%, respectively^61,62^. The positive correlation between N supply and protein content in many tissues of different species has been shown previously^63^. We measured the total protein content (see Supplementary Information) in all segments and observed that, with the exception of B0, high-N supply increased the total protein content, which was highest in the M and T segments in both genotypes (see Supplementary Fig. S7). Additionally, the total N content showed the same trend (Fig. 3b). Interestingly, genotype was not considered a factor that influenced the ability to accumulate N along the leaf (p-value: 0.361). For instance, despite the lower N content in the M segment of the R genotype (Fig. 3b), this genotype had lower Δ^13^C discrimination (Fig. 3a). In a previous report^39^, the M and T leaf segments of sugarcane cultivated without differential N conditions had higher N contents than did the other segments. Therefore, because the M and T segments had the highest levels of protein (Supplementary Fig. S7), total N (Fig. 3b) and chlorophyll (Fig. 2), our data confirm that these leaf regions are more photosynthetically active than B0 and B.

**Figure 4:**
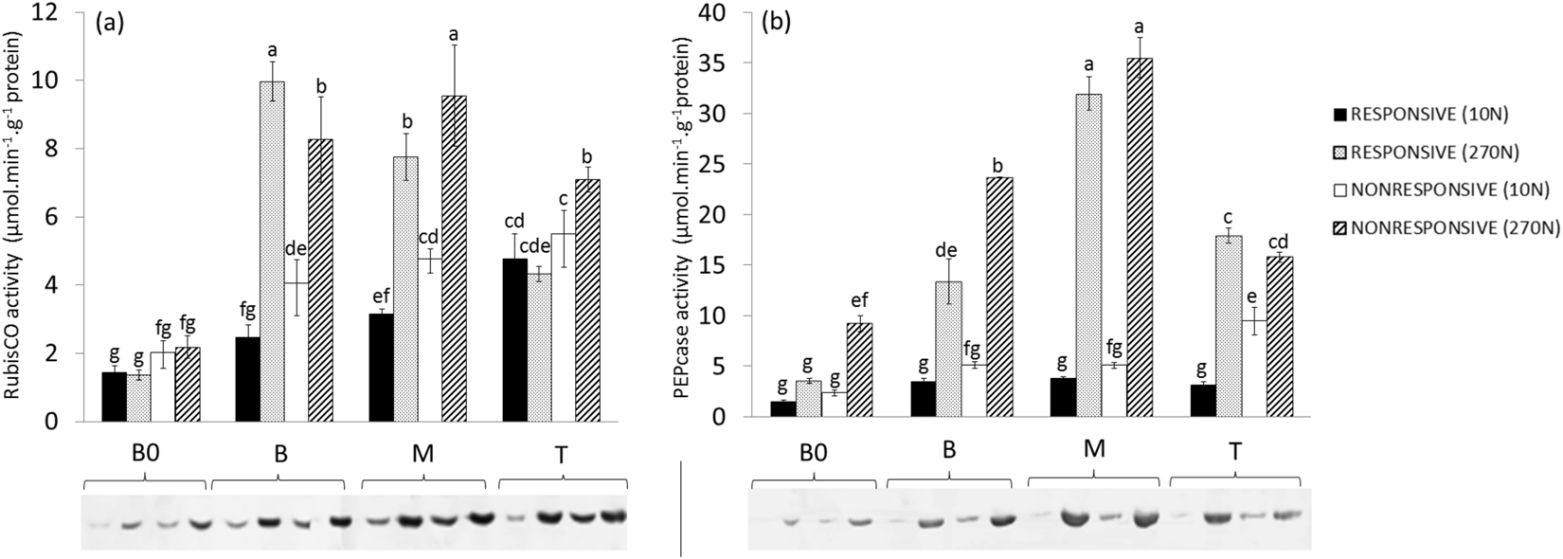
Enzyme activity and the level of carboxylation enzymes along the sugarcane leaf blade. (**a**) Ribulose-1,5-bisphosphate carboxylase/oxygenase (RubisCO); (**b**) Phosphoenolpyruvate carboxylase (PEPcase). The commercial peptide sequences of RubisCO large subunit form I and PEPC1 (Agrisera, SWE) were used. The cropped gel image corresponds to a western-blot assay representative of three independent experiments. The full-length gels are presented in Supplementary Figs 8 and 9. B0, Base “zero”; B, Base; M, Middle; T, Tip. 10 N and 270 N correspond to treatments of 10 and 270 mg of N per kg of sand, respectively. Data are presented as the mean ± SE. Letters indicate statistical significance using ANOVA followed by Fisher’s exact test (n = 3; P ≤ 0.05).

The 270 N treatment also increased the amount and activity levels of carboxylation enzymes along the sugarcane leaf blade in both genotypes (Fig. 4), corroborating other studies of sugarcane^17^ and other grass species^20,64^. N-deficient maize hybrids showed a decrease in PEPcase activity under field conditions^65^. Sun^20^ observed that RubisCO activation was lower in rice plants cultivated under low-N conditions than in those cultivated under high-N conditions. In sugarcane, N-deficient plants also presented reduced partitioning of the carboxylase activity of RubisCO and PEPcase^17^.

As shown in Fig. 4, the increase in the activity and amount of RubisCO and PEPcase was more pronounced in the segments located in the middle leaf region (B and M segments). Although one study has shown that RubisCO activity decreases to a greater extent than PEPcase in sugarcane plants cultivated under N-limiting conditions^6^, our data showed that N supply caused a more relevant increase in PEPcase activity (Fig. 4b) than in RubisCO activity (Fig. 4a). This result suggests that the response of PEPcase activity to N supply was greater than that of RubisCO activity in sugarcane plants.

### **Metabolic profile varies** according **to leaf segment, genotype and N supply**

**Figure 5:**
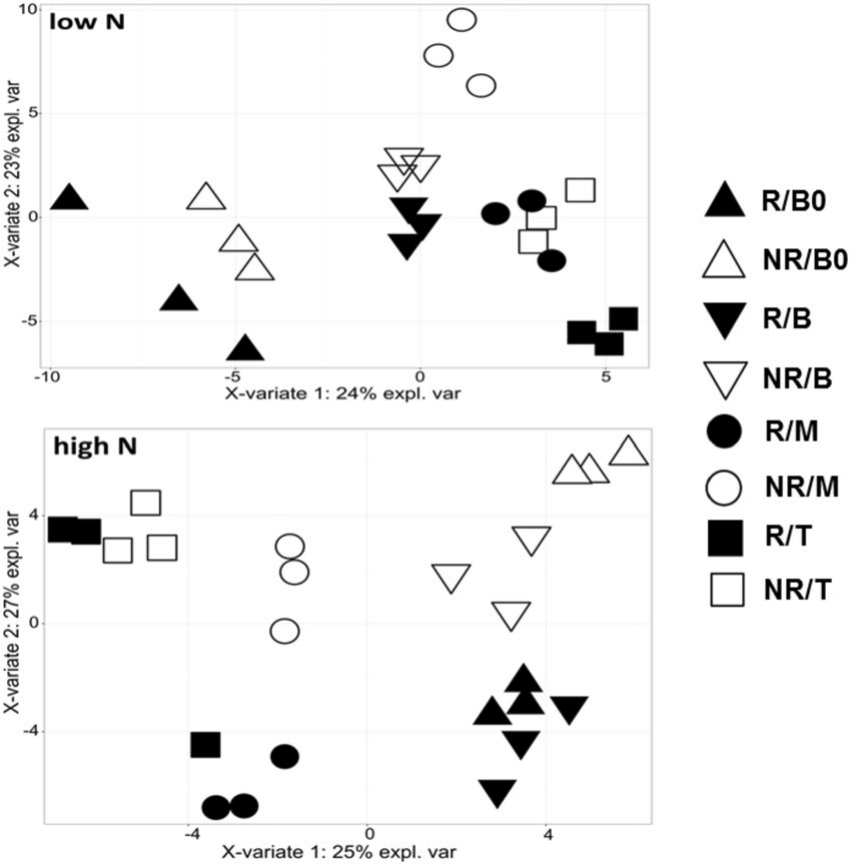
Results of partial least square discriminant analysis (PLS-DA) in relation to metabolites detected by GC-MS. low N corresponds to the treatment of 10 mg of N per kg of sand; high N corresponds to the treatment of 270 mg of N per kg of sand; R, responsive genotype; NR, nonresponsive genotype; B0, Base “zero”; B, Base; M, Middle; T, Tip.

Untargeted analysis of metabolite profiles determined by GC-TOF-MS identified a total of 67 metabolites composed primarily of proteinogenic amino acids and carbohydrates (see Supplementary Fig. S10). The four leaf segments from both genotypes differed in their metabolic profile, as evidenced by PLS-DA analysis (Fig. 5). In contrast to PCA, which treats all variables the same way, PLS-DA analysis treats one variable as the dependent variable and considers relationships with that variable, maximizing the separation of classes^66,67^, which is ideal in a classification scenario.

Under high-N conditions, differences between genotypes were greater than those under low-N conditions (Fig. 5). The second component (accounting for 27% of the variation) better separated the segments within genotypes under high-N conditions, indicating that the two genotypes presented distinct metabolic changes along the leaf blade in relation to N supply. Under the same conditions, clusters of B0 and B formed by PLS-DA analysis were closest to each other and distant from the M and T clusters, indicating that there were metabolic differences in the leaf regions towards the B and T regions and that these differences were increased due to the N supply. The plot of PLS-DA analysis with all samples can be found in Supplementary Fig. S11.

Other studies of grass species have also shown differences in the metabolic profile among different leaf segments. Pick *et al*.^32^ used PCA of transcripts and metabolites observed a clear separation of the slices along the maize leaf, with greatest differences between basal and tip regions. Czedik-Eysenberg *et al*.^33^ verified that the metabolite profile related to carbon metabolism differs between different zones of maize leaves, being more divergent amongst regions rich in dividing cells and in mature zones. Metabolites associated with photosynthesis and N metabolism showed opposite trends in the distal regions of maize and rice leaves^35^. The differences in the metabolite profiles that we observed between sugarcane leaf segments under low- and high-N conditions, combined with other differences in chlorophyll, protein, and sugar contents and in the activity and levels of carboxylation enzymes, confirm the existence of a developmental gradient along grass leaves^32–34^, including those of sugarcane^39^. However, sugarcane responses are dependent not only on the leaf region and N supply but also on the genotypes’ responsiveness to N treatment.

**Figure 6:**
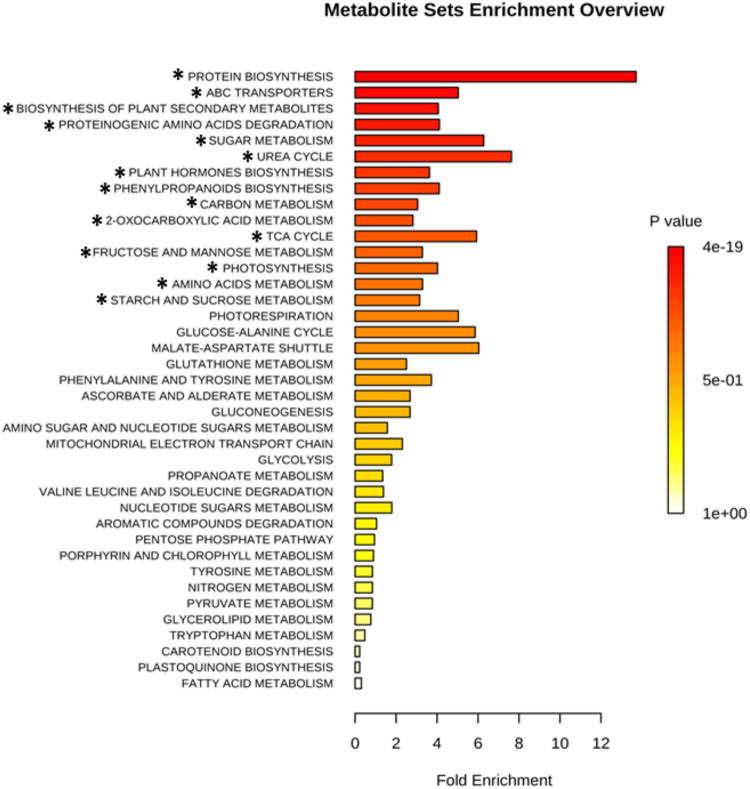
Summary plot for the over representation analysis (ORA) of metabolic pathways. The fold enrichment was calculated among all metabolites detected (67) and the metabolites present in each pathway of the customized library. The *p* values are colour-coded, with dark red being highly significant and white being least significant. (*) Significant pathway at the P < 0.05 level and FDR ≤ 0.1.

MSEA (metabolite set enrichment analysis) with over representation analysis (ORA) approach was done in order to identify in which pathways the detected metabolites were related. In this analysis, no quantitative data are used, and a list of the identified metabolites is provided. Therefore, the fold enrichment was calculated among all the 67 metabolites detected and the metabolites present in each pathway of a customized library containing 39 pathways. For example, considering the photosynthesis pathway with 32 metabolites (Table S2) and the list of detected metabolites (67), a random value of approximately 2 would be expected, but the ORA showed 8 metabolites with 5% of significance. Out of the 39 pathways in the analysis, 15 were considered significant (Fig. 6), including primary and secondary plant metabolism pathways. Based on the high number of proteinogenic amino acids (20), protein biosynthesis was the most enriched pathway (p-value = 1.43 × 10^−17^; FDR = 1.43 × 10^−17^). Furthermore, considering the high number of carbohydrate metabolites (28), pathways related to energetic and sugar metabolism were also enriched, such as photosynthesis (p-value = 1.29 × 10^−2^; FDR = 1.42 × 10^−3^), carbon metabolism (p-value = 1.76 × 10^−4^; FDR = 2.44 × 10^−5^), and starch and sucrose metabolism (p-value = 1.86 × 10^−2^; FDR = 1.91 × 10^−3^). The p-values and FDR of all metabolic pathways can be found in Supplementary Table S2.

**Figure 7:**
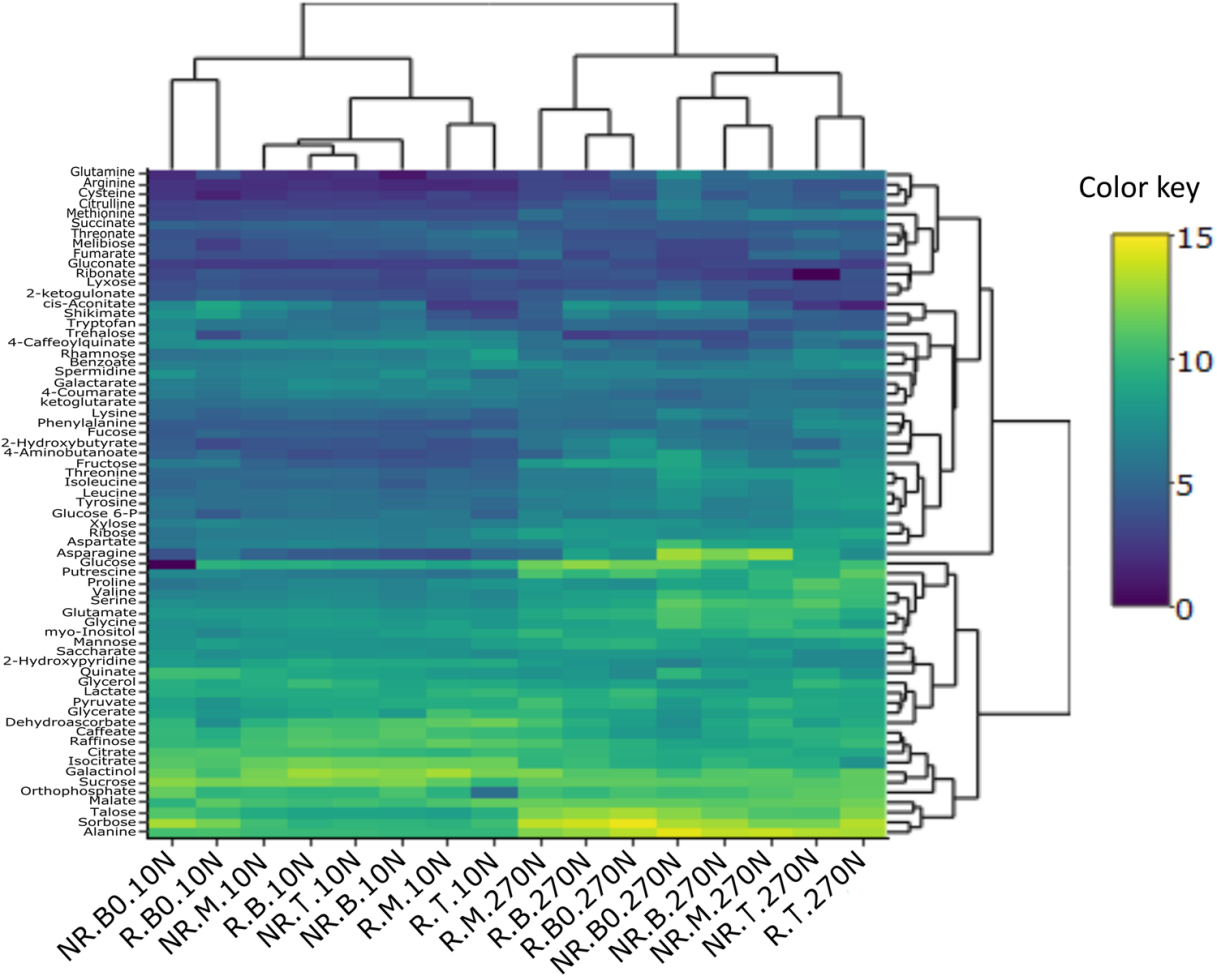
Hierarchical cluster analysis coupled with a heat map showing metabolite profiles of different segments along sugarcane leaf blade. The ratio of metabolite abundance is represented as the relative concentration in relation to the total ion count (TIC). The intensity of each metabolite was normalized to both by dividing the dry weight of the sample and by the sum of the total ion counts. To further correct for the measurement effects, values were then normalized by the median of the all measured data and log2 transformed. 10 and 270 correspond to treatments of 10 and 270 mg of N per kg of sand, respectively. R corresponds to responsive genotype while NR corresponds to nonresponsive genotype. B0, Base “zero”; B, Base; M, Middle; T, Tip. The colour key represents the relative concentration of metabolites.

The hierarchical clustering of metabolites showed that N supply was able to modify the metabolite profile, separating the samples into two main clusters (Fig. 7, horizontal axis). Additionally, as in the discriminant analysis results (Fig. 5), in the 270 N treatment, the clustering of the segments separated better between the R and NR genotypes, with the only exception being the tip segments. High N supply markedly increased the levels of several amino acids (e.g., tyrosine, threonine, alanine, serine, proline and asparagine (Asn)) along the sugarcane leaf blade (Fig. 7), which indicates the direct influence of N in protein biosynthesis. Curiously, this trend was not observed for tryptophan (Trp). Increased levels of amino acids were also correlated with a higher total protein content in high-N plants (see Supplementary Fig. S7). In maize, wheat and tomato plants, N deficiency caused a significant decrease in amino acid levels^23,68,69^. On the other hand, low-N supply caused an increase in some amino acids levels in different accessions of Arabidopsis^70,71^. These authors associated this increase with the ability to adjust the biosynthesis of amino acids in a coordinate manner to compensate for the decrease in N supply.

The leaf segments and genotypes displayed characteristic changes in relation to the levels of amino acids. The amounts of tyrosine, threonine and serine increased at the T segment compared to other segments of both genotypes. The same trend was observed in different regions of rice leaves^35^. In contrast, the level of these amino acids decreased significantly between the B and T of wheat leaves in plants treated with nitrate^23^ and in maize leaves from plants grown without N limitation^33,35^. The metabolites also clustered according to their changes in abundance between the samples (Fig. 7, right vertical axis). For example, Asn, a N-rich amino acid, showed the most distinct profile in comparison to the other metabolites. It was most abundant in the B0, B and M segments of the NR genotype in the higher N treatment. In addition, glutamate (Glu) and glutamine (Gln), also had higher values in the same segments in the NR genotype, suggesting that the ammonium assimilation to glutamic acid is greater in this genotype or that the senescence process is more active in the leaves of this genotype. A metabolic model of N assimilation and remobilization proposed by Masclaux-Daubresse *et al*.^24^ shows that Gln is mainly synthetized in senescing leaves and that the Glu levels increase due to a series of transamination reactions using the amino acid pool released via the proteolysis of chloroplast proteins. Furthermore, in sunflower, rice and Arabidopsis, the Asn levels increased during leaf senescence^72–74^. However, to confirm this hypothesis, more studies related to activity, transcript expression level and post-translational regulation of the two main enzymes related to ammonium assimilation (Gln synthetase, GS and Glu oxoglutarate aminotransferase, GOGAT) and the enzymes induced during leaf senescence (cytosolic Gln synthetase, GS1; Glu dehydrogenase, GDH and Asn synthetase, AS)^71,73^ are needed.

As shown in Fig. 7, alanine (Ala) accumulated to higher levels in all segments of the R and NR genotypes in the 270 N treatment than did the other amino acids. The N supply also increased Asp levels along the leaf. Several studies with C_4_ species have shown that Asp and Ala can carry part of the carbon flow in the canonical NADP-ME C_4_ pathways between mesophyll and bundle sheath cells^75,76^. Furthermore, labelling experiments have indicated that as much as 25% of the carbon first labels Asp, not malate^77^. Pick *et al*.^32^ quantified Ala and Asp, and after integrating with transcriptomic data from different maize leaf segments, they concluded that the core C_4_ cycle in maize is branched, with two C_4_ acids, including Asp, and three C_3_ acids. Moreover, these acids are responsible for carbon distribution, which reduces the diffusion requirements between the mesophyll and bundle sheath for any single molecule. Our data indicate that same occurs in sugarcane plants via N regulation.

Certain phenylpropanoids, such as 4-coumarate, quinate and 4-caffeoylquinate, tended to increase in all leaf segments in plants from both genotypes grown under low-N conditions (Fig. 7). In leaves of maize plants cultivated under N-deficient conditions, an increase in the concentration of the same compounds was also shown^69^. Our results suggest that N supply inhibits the biosynthesis of some phenylpropanoids that are involved in lignin biosynthesis and other large sectors of secondary metabolism in sugarcane leaves^78^.

In addition to the phenylpropanoid content, the amount of organic acids from the TCA cycle also differed along the sugarcane leaf and between N treatments. Among 12 compounds present in the TCA cycle, 8 were identified in our study. With the exception of malate and pyruvate, a decrease in the majority of organic acids occurred in plants treated with 270 N, particularly in sections near the tip region (M and T segments). This decrease may be related to the use of organic acids for ammonium assimilation, which results in a depletion of these compounds^79,80^. Allwood *et al*.^23^ noted that the highest concentration of these organic acids occurred in the B segment of wheat leaves under high-nitrate conditions and decreased towards the tip, where the leaf is more photosynthetically developed. We deduce that the higher content of these organic acids towards the base, as evidenced in other grass species such as wheat and maize^23,33^, is indicative of higher respiration rate in the youngest tissue of sugarcane leaf. However, this pattern seems to vary in other species. For example, a reduction in the content of organic acids from the TCA cycle (e.g., fumarate, citrate and isocitrate) was reported in leaves from Arabidopsis plants treated with different N levels, but this reduction was greater in plants treated with low N levels^70,71^. In addition, a reduction in most TCA cycle organic acids was seen in N-deficient tomato and maize leaves^68,69^.

**Figure 8:**
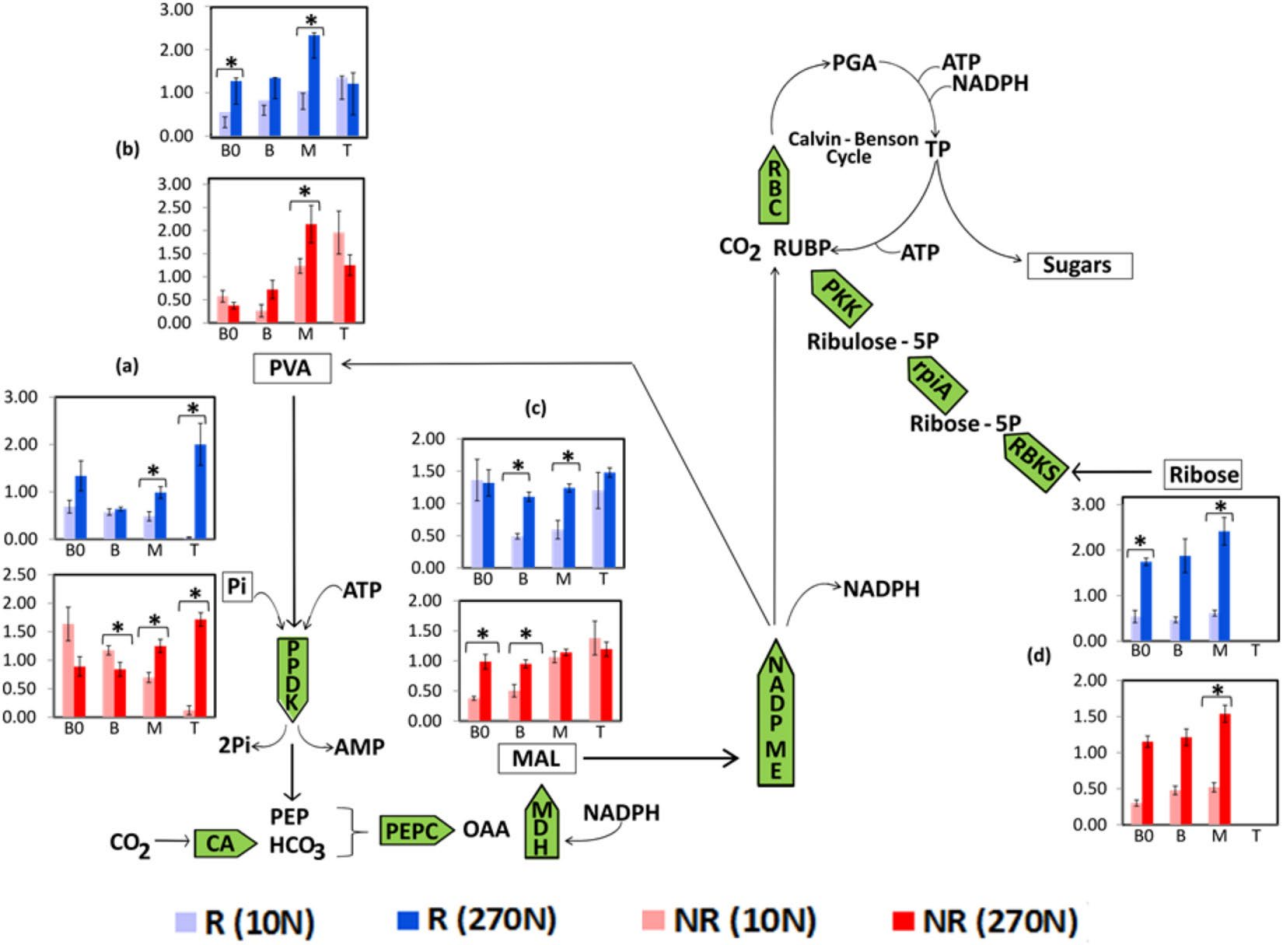
Scheme of the sugarcane photosynthetic pathway showing the quantification of phosphate (**a**), pyruvate (**b**), malate (**c**) and ribose (**d**) along the sugarcane leaf blade. ATP, adenosine triphosphate; CA, carbonic anhydrase; MDH, malate dehydrogenase; NADPH, nicotinamide adenine dinucleotide phosphate, NADP ME, NADP malic enzyme; OAA, oxaloacetate; PEP, phosphoenolpyruvate; PEPC, PEP carboxylase; PGA, phosphoglycerate; PKK, ribulose-5-phosphate kinase; PPDK, pyruvate Pi-dikinase; RBC, Rubisco; RUBP, Ribulose-1,5-bisphosphate; TP, triose phosphates; RBKS, ribokinase; rpiA, ribose 5-phosphate isomerase. R corresponds to the responsive genotype, and NR refers to the nonresponsive genotype. B0, Base “zero”; B, Base; M, Middle; T, Tip. Note: The ratio of metabolite abundance is represented by relative concentration in log_2_ scale. Data are presented as the mean ± SE with five replications. (*) indicates values determined by the Student’s t-test to be significantly.

Metabolites related to photosynthesis were also altered along the sugarcane leaf blade. Previous studies reported an increase in some photosynthesis-related compounds in association with N supply in different regions of the maize leaf, such as malate and pyruvate^32,35^. In the sugarcane leaf, these alterations were significantly different in the R and NR genotypes (Fig. 8). The middle leaf regions in the R genotype, represented by B and M segments, showed a higher increase in malate (Fig. 8c) and pyruvate (Fig. 8b) under high-N supply than did these regions in the NR genotype. Moreover, the B segment of the NR genotype exhibited a significant decrease in the concentration of phosphate, ranging from 1.17 (10 N) to 0.84 (270 N) (Fig. 8a), while pyruvate (Fig. 8b) had lower levels in the B0 and T segments, varying from 0.58 to 0.38 and from 1.96 to 1.25, respectively. In addition, the levels of ribose, a substrate of RubisCO, increased along leaf segments in response to N (Fig. 8d), consistent with RubisCO quantification analysis (Fig. 4a). Furthermore, the ribose levels were higher in the R than in the NR genotype (Fig. 8d). Considering the alterations in photosynthesis-associated metabolites, we suggest that N positively affects the establishment of photosynthesis and that this affect is more pronounced in the M leaf region, where photosynthesis is more active, than in the B and T regions, as described in a previous study^39^. In addition, in contrast to the NR genotype, the elevated amount of metabolites related to photosynthesis quantified in almost all segments of the R genotype under high-N conditions suggests a more intense metabolic response in this genotype.

### Differential N supply causes alterations in soluble sugar levels along the leaf blade

The concentration of soluble sugars in sugarcane leaves is used to evidence biochemical and physiological alterations associated with biotic^81^ and abiotic^82^ stresses that lead to nutrient deprivation, including N deprivation^83^. Furthermore, leaf sugar content is a parameter to assess the source-sink relationship between tissues based on the consumption and export rates and the regulation of photosynthesis by sugars^84,85^.

The content of some soluble sugars measured by metabolomics also varied with N supply, leaf segment and genotype. In plants supplied with high N levels, glucose and fructose were detected in higher quantities in the B0 and B segments than in the M and T segments, with greater accumulation in the R genotype (Table 1). In contrast to our data, Allwood *et al*.^23^ observed in wheat that fructose levels were higher in the T of the leaves than in the M and B regions in plants supplemented with nitrate. Mattiello *et al*.^39^, using another genotype, found a clear trend of increased levels of glucose and fructose towards the T segment in sugarcane plants cultivated without N limitation. However, other studies also verified an increase in glucose and fructose next to the leaf B region. Wang *et al*.^35^ and Pick *et al*.^32^ analysed the metabolic profile of different maize leaf segments and showed that glucose and fructose levels were higher in regions close to the base and that these levels decreased along the leaf blade towards the tip. An explanation for the differential accumulation of simple sugars along the sugarcane leaf blade is related to the specific characteristics of leaf development in this species. Considering that grass leaves have basipetal development^86^ with a cellular developmental gradient along the leaf blade^32,34^, cellular replication is concentrated in the B region, where the demand for energy provided by simple sugars is higher. Therefore, the net carbon reserve varies among grass species, leaf regions and N conditions.

**Table 1:** Relative concentration of soluble sugars detected by GC-MS in different sugarcane leaf segments. The ratio of metabolite abundance is represented as the relative concentration in relation to the total ion count (TIC). The intensity of each metabolite was normalized to both by dividing the dry weight of the sample and by the sum of the total ion counts. To further correct for the measurement effects, values were then normalized by the median of the all measured data and log2 transformed. B0, Base “zero”; B, Base; M, Middle; T, Tip. 10 N and 270 N correspond to treatments of 10 and 270 mg of N per kg of sand, respectively. Note: Values are presented as the mean ± SD with three replications and referred. Letters indicate statistical significance using ANOVA followed by Fisher test (n = 3; P ≤ 0.05).

Genotype/Segment	Glucose	Fructose	Sucrose	Raffinose	Galactinol	Rhamnose	Trehalose
**Treatment = 10 mg of N**							
Responsive / B0	9.65 ± 1.30^bcd^	5.47 ± 1.63^efg^	12.04 ± 0.68^ab^	10.44 ± 1.43^bcd^	11.14 ± 1.14^efg^	5.92 ± 0.84^d^	4.25 ± 1.44^de^
Nonresponsive / B0	—	6.12 ± 0.77^de^	12.42 ± 0.16^a^	10.09 ± 0.24^de^	11.47 ± 0.16^def^	5.55 ± 0.28^de^	6.9 ± 1.00^a^
Responsive / B	9.22 ± 0.24^d^	3.81 ± 0.68^hi^	12.24 ± 0.13^a^	11.13 ± 0.12^ab^	12.70 ± 0.29^a^	5.87 ± 0.43^d^	5.36 ± 1.29^bcd^
Nonresponsive / B	9.03 ± 0.34^d^	3.61 ± 0.73^i^	12.14 ± 0.08^a^	10.94 ± 0.27^abc^	12.34 ± 0.33^abc^	6.12 ± 0.36 ^cd^	6.85 ± 0.68^a^
Responsive / M	8.50 ± 0.42^d^	3.76 ± 0.51^hi^	11.25 ± 0.64^c^	11.23 ± 0.60^ab^	12.77 ± 0.60^a^	6.71 ± 0.54^bc^	6.07 ± 1.48^abc^
Nonresponsive /M	9.05 ± 0.22^d^	3.63 ± 0.82^i^	11.40 ± 0.24^c^	11.21 ± 0.19^ab^	12.50 ± 0.06^ab^	7.2 ± 0.50^b^	6.28 ± 0.54^abc^
Responsive / T	8.94 ± 0.56^d^	4.59 ± 0.56^ghi^	10.04 ± 0.87^d^	11.31 ± 0.73^a^	12.39 ± 0.77^ab^	8.62 ± 0.55^a^	6.41 ± 1.04^ab^
Nonresponsive / T	8.78 ± 0.76^d^	4.84 ± 0.88^fgh^	10.20 ± 0.82^d^	10.64 ± 0.7^bcd^	11.71 ± 0.74^cde^	8.71 ± 1.12^a^	5.3 ± 1.33^bcd^
**Treatment = 270 mg of N**							
Responsive / B0	11.98 ± 0.59^a^	8.89 ± 0.66^a^	11.45 ± 0.31^bc^	9.91 ± 0.81^def^	11.23 ± 0.37^ef^	6.01 ± 1.02 ^cd^	3.4 ± 0.49^ef^
Nonresponsive / B0	11.21 ± 0.93^a^	8.12 ± 1.28^abc^	10.95 ± 0.39^c^	8.22 ± 0.81 ^g^	9.99 ± 0.48^i^	4.87 ± 0.14^e^	3.36 ± 1.09^ef^
Responsive / B	11.99 ± 1.14^a^	8.23 ± 1.09^ab^	11.28 ± 0.09^c^	10.04 ± 0.32^de^	10.96 ± 0.47^fgh^	4.83 ± 0.76^e^	2.9 ± 0.74 ^f^
Nonresponsive / B	10.28 ± 1.04^b^	7.05 ± 1.15 ^cd^	11.02 ± 0.43^c^	8.25 ± 0.39 ^g^	10.51 ± 0.63^ghi^	4.81 ± 0.13^e^	3.25 ± 0.62^ef^
Responsive / M	11.51 ± 0.37^a^	7.96 ± 0.60^abc^	11.18 ± 0.25^c^	10.56 ± 0.34^abc^ c	11.98 ± 0.23^bcd^	5.61 ± 0.33^d^	5.24 ± 0.56^bcd^
Nonresponsive / M	9.23 ± 0.32 ^cd^	5.85 ± 0.95^ef^	11.14 ± 0.31^c^	9.22 ± 0.59 ^f^	11.42 ± 0.57^def^	5.91 ± 0.26^d^	5.12 ± 0.72 ^cd^
Responsive / T	10.09 ± 0.59^bc^	7.34 ± 0.41^bc^	11.34 ± 0.20^c^	10.22 ± 0.85^cde^	11.45 ± 0.40^def^	7.19 ± 0.62^b^	6.38 ± 0.60^ab^
Nonresponsive / T	9.48 ± 0.31^bcd^	6.16 ± 0.17^de^	11.03 ± 0.46^c^	9.59 ± 0.30^ef^	10.35 ± 0.31^hi^	7.24 ± 0.27^b^	5.87 ± 0.26^abc^

Interestingly, under low-N conditions the levels of some sugars, such as raffinose, galactinol, rhamnose and trehalose, were higher than those under high-N conditions in almost all segments along the sugarcane leaf blade (Table 1). For example, in the B segment in the 10 N treatment compared to the 270 N treatment, trehalose levels were 84.82% and 110.76% higher in the R and NR genotypes, respectively. Several studies have also reported an increase in these molecules in leaves of other species cultivated under low-N conditions^69,70,87^. Consistent with our data, Allwoood *et al*.^23^ also verified a decrease in trehalose content in the basal region of wheat leaves treated with high nitrate concentrations. These sugars were previously described as signalling molecules for other kinds of abiotic stresses in plants, such as extreme temperatures and drought^83,88^. In *Arabidopsis thaliana*, raffinose acts as an osmoprotectant under drought conditions^49^. In maize, compounds of the raffinose family are considered drought-related metabolites^32^. The full role of raffinose in sugarcane is still a matter of speculation, but some studies have shown that it is correlated with storage compounds, such as sucrose, and signalling compound products^30^.

Therefore, we suggest that the response of some signalling sugars to N-deficient conditions in sugarcane leaf is similar to that observed to other abiotic stresses.

## Conclusions

The influence of N on photosynthesis establishment and other metabolic pathways related to growth and leaf development metabolism is highly dynamic and evidenced through differences in several traits evaluated along the sugarcane leaf and between R and NR genotypes. N supply increases the photosynthetic apparatus along the sugarcane leaf by increasing chlorophyll content, the amount and activity of carboxylation enzymes, total protein, sugar content, total N and photosynthesis-related metabolites. In contrast to the B region, with a high respiration rate and energy demand, the M leaf region had a more developed photosynthesis and a more active metabolic state, which consequently increased the demand for N. In addition, metabolite traits indicated that the carbon distribution of core C_4_ cycle is branched with two C_4_ acids (Asp and Ala) under high N, as already demonstrated in maize leaves. In relation to differences between genotypes, the R genotype showed less evidence of senescence in the T region and a more efficient carboxylation process mediated by PEPcase along the entire leaf, in addition to higher levels of photosynthesis-related compounds. Therefore, the pathways modified by N presented in this study can be considered targets for further studies to understand and improve the response of sugarcane to N supply, aiming to increase photosynthesis and, consequently, yield.

## Electronic supplementary material

Supplementary Information
