# Supplementary material for: Nitrogen supply influences photosynthesis establishment along the sugarcane leaf
Source: Sci Rep. 2018 Feb 2;8:2327. doi: 10.1038/s41598-018-20653-1 (PMC5797232; doi:10.1038/s41598-018-20653-1)
Supplement: Supplementary file 1 — Supplementary Information [file 41598_2018_20653_MOESM1_ESM.pdf]

Supplementary Information

Nitrogen supply influences photosynthesis establishment along the sugarcane leaf

Authors: Denis Bassi[1] (denis@lgf.ib.unicamp.br), Marcelo Menossi[1] (menossi@lgf.ib.unicamp.br) and Lucia Mattiello[1] (lucia@lgf.ib.unicamp.br)

1 Departamento de Genética, Evolução e Bioagentes, Instituto de Biologia, Universidade Estadual de Campinas, 13083-862, Campinas, Brasil

Supplementary Methods

Biometric measurements (number of tillers, number of leaves, number of green leaves, number of drought leaves, culm diameter, leaf +1 height, SPAD and foliar area) drought biomass (root and shoot) and physiological parameters (photosynthesis, stomatal conductance, internal CO2 concentration, electron transport rate, photochemical efficiency of PSII and transpiration), totalling 17 traits, were submitted to principal component analysis (PCA) using the mixOmics R package[1] in order to validate the contrasting response of genotypes in relation to N supply.

Leakiness was calculated according to the formula described by Farquhar[2] and was determined in the M segment. Total protein content was determined with Bradford solution (0.25%). Absorbance readings were measured in a microplate spectrophotometer (Infinite 200 PRO NanoQuant, Tecan, CH), and readings were taken at a wavelength of 595 nm.

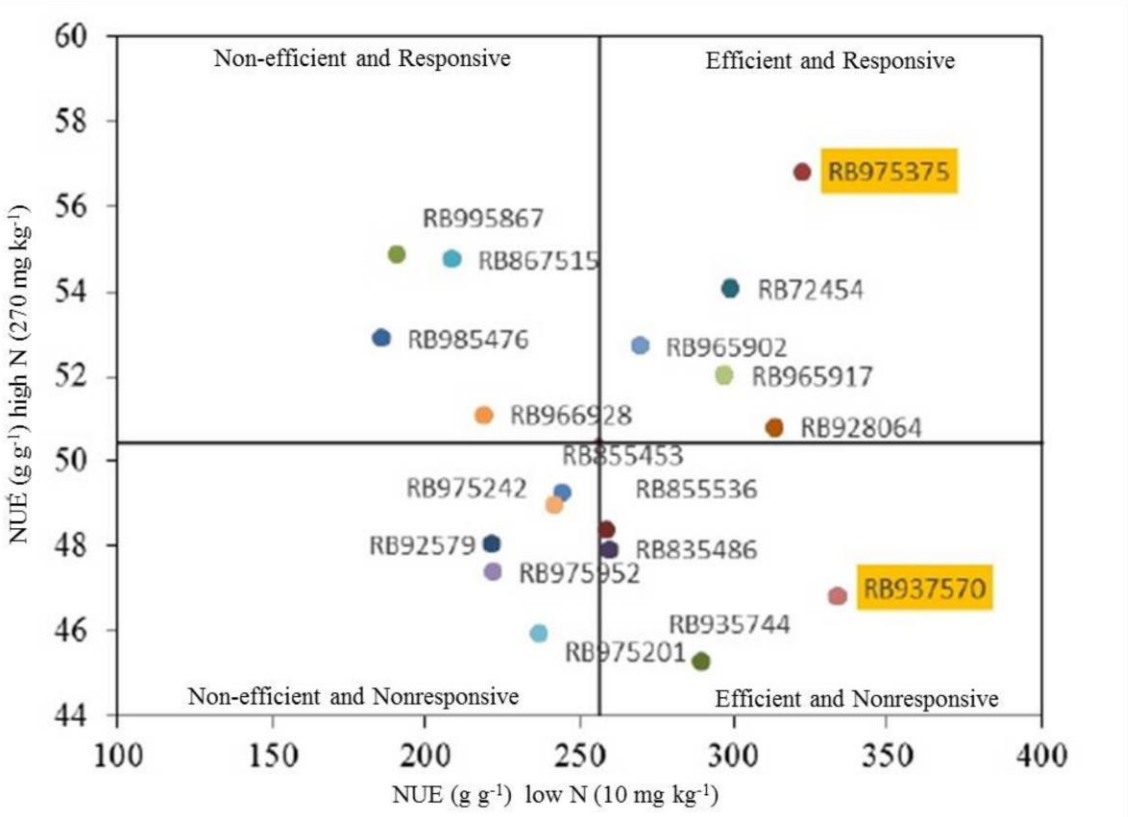

Supplementary Figure S1. Screening of 20 sugarcane genotypes using NUE (nitrogen use efficiency) analysis based on methodology developed by Robinson[3]. NUE was determined by dividing biomass yield by crop N uptake. The responsiveness of genotypes was determined according to the threshold (NUE ≈ 50 g g-1 under high-N conditions) used in the screening analysis. The nonresponsive genotypes presented NUE < 50 and responsive genotypes NUE ≈ 50.”

Supplementary Table S1. Content of macronutrients and micronutrients used in the fertirrigation solution.

	Macronutrients					Micronutrients						
	$H_2PO_4^-$	K^+	Ca^{2+}	Mg^{2+}	SO_4^-	H_3BO_3	Cu^{2+}	Fe^{2+}	Zn^{2+}	Mn^{2+}	MoO_4^{2-}
Content (mg per kg of sand)	100	100	20	25	100	1	0.5	5	3	4	0.3

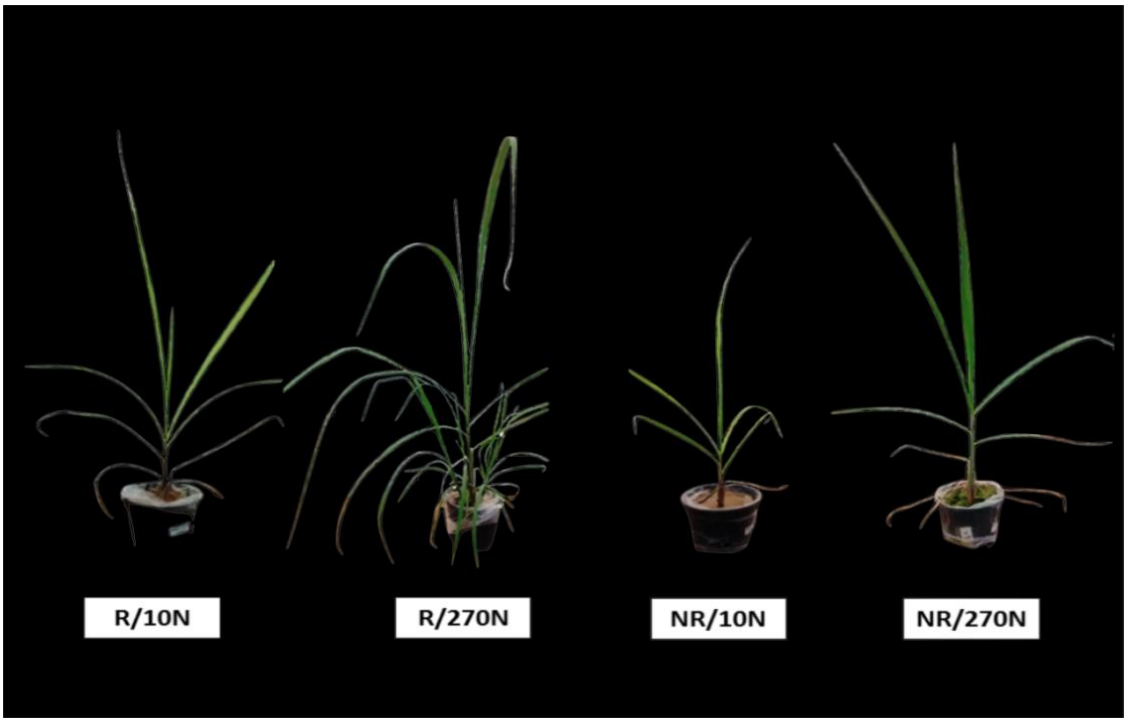

Supplementary Figure S2. **Three-month-old sugarcane plants.** R corresponds to the responsive genotype and NR to the nonresponsive genotype; 10N and 270N indicate 10 and 270 mg of N per kg of sand treatments, respectively.

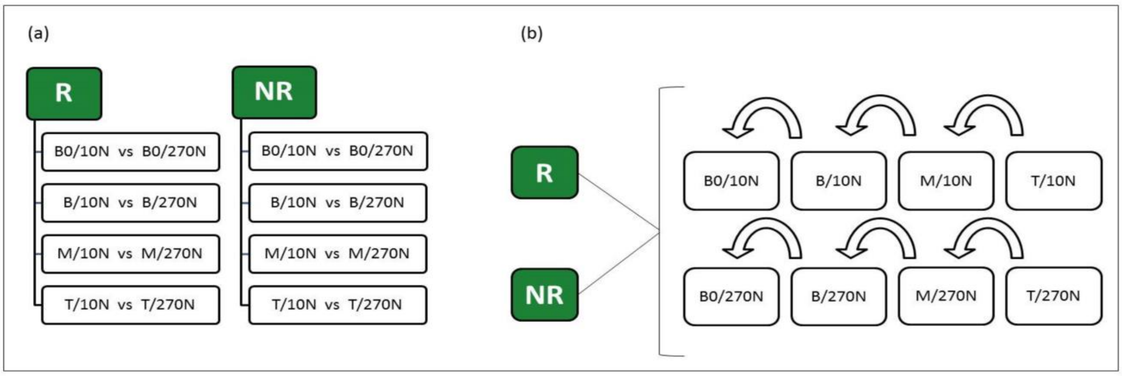

Supplementary Figure S3. Schematic of the strategies used for t-test analysis applied to metabolomics data. (a) Comparisons between treatments for each segment within each genotype; (b) Comparisons among distal segment to the previous segment within each leaf and each treatment for each genotype; R corresponds to the responsive genotype and NR to the nonresponsive genotype; 10N and 270N correspond to 10 and 270 mg of N per kg of sand treatments, respectively; B0, Base “zero”; B, Base; M, Middle; T, Tip.

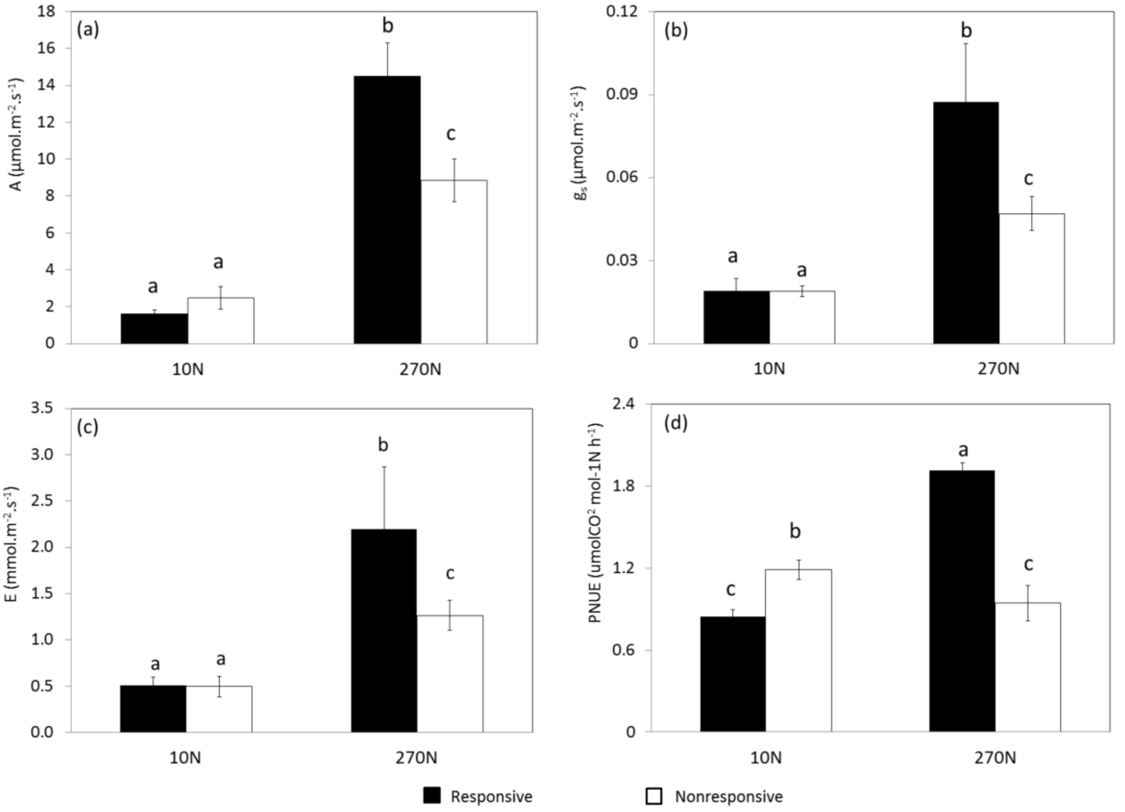

Supplementary Figure S4: Effects of different N treatments on physiological parameters of two contrasting genotypes. Physiological data were collected at middle leaf region of leaf +1 from three-month old plants. (a) Leaf photosynthetic rate; (b) Stomatal conductance rate; (c) Transpiration rate; (d) PNUE, photosynthetic nitrogen-use efficiency; 10N and 270N correspond to 10 and 270 mg of N per kg of sand, respectively. Data are presented as the mean ± SE. Letters indicate statistical significance using ANOVA followed by Fisher test ($n = 3$; $p \leq 0.05$).

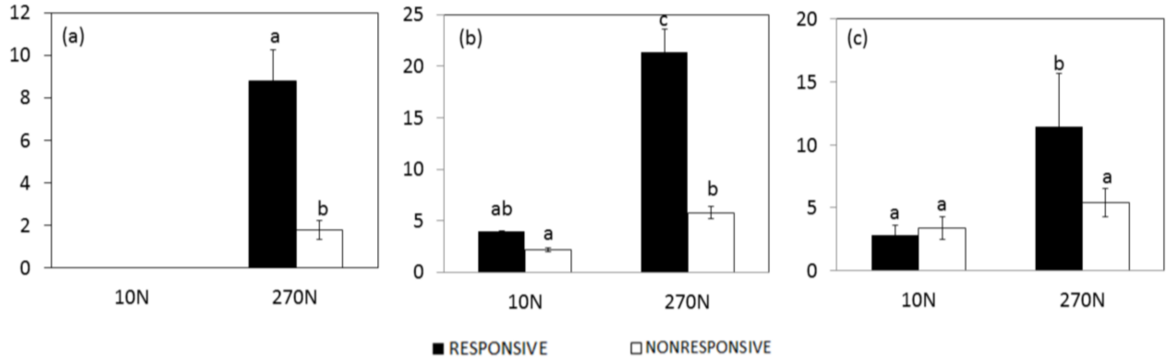

Supplementary Figure S5: Effects of different N treatments on biometric traits. (a) tillering; (b) number of green leaves; (c) number of dry leaves. 10N and 270N correspond to 10 and 270 mg of N per kg of sand, respectively. Data are presented as the mean ± SE. Letters indicate statistical significance using ANOVA followed by Fisher test ($n = 3$; $p \leq 0.05$).

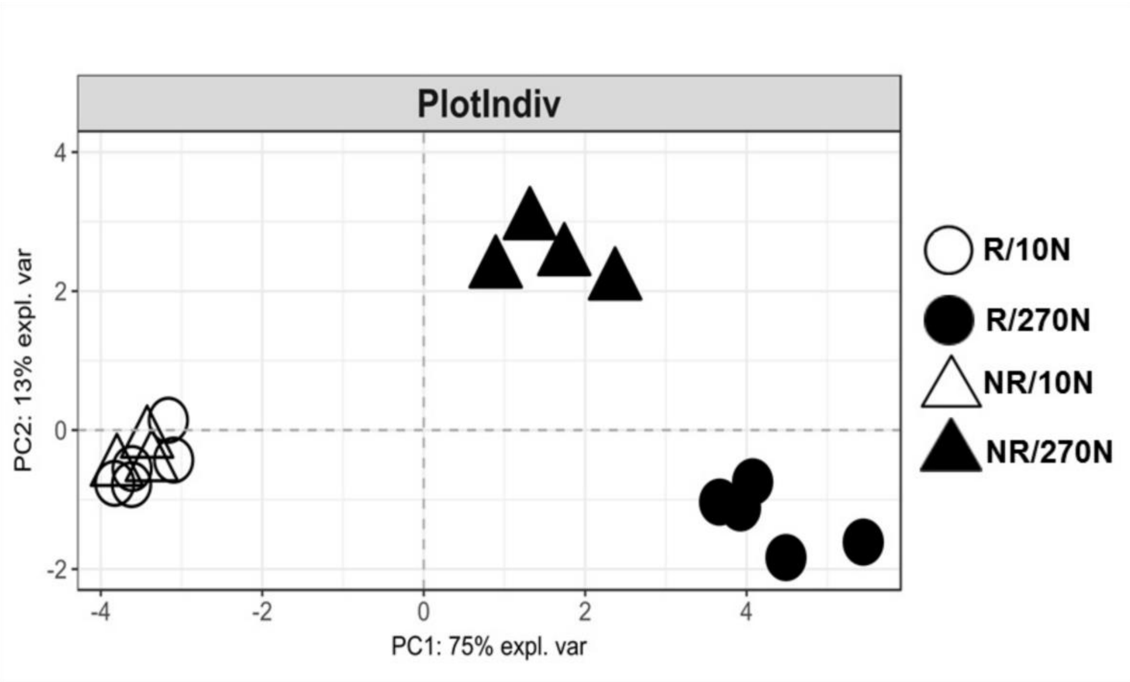

Supplementary Figure S6: Multivariate PCA analysis using two principal components. A total of 17 traits were used: tillering, total number of leaves, number of green leaves, number of dry leaves, culm diameter, height of leaf +1, SPAD index, leaf area, dry leaf biomass, dry culm biomass, dry root biomass, photosynthesis (A), stomatal conductance, internal carbon concentration (Ci), electron transport rate (ETR), potential quantum efficiency of photosystem II (Fv/Fm) and transpiration rate. 10N and 270N correspond to 10 and 270 mg of N per kg of sand, respectively. R, responsive (RB975375). NR, nonresponsive (937570).

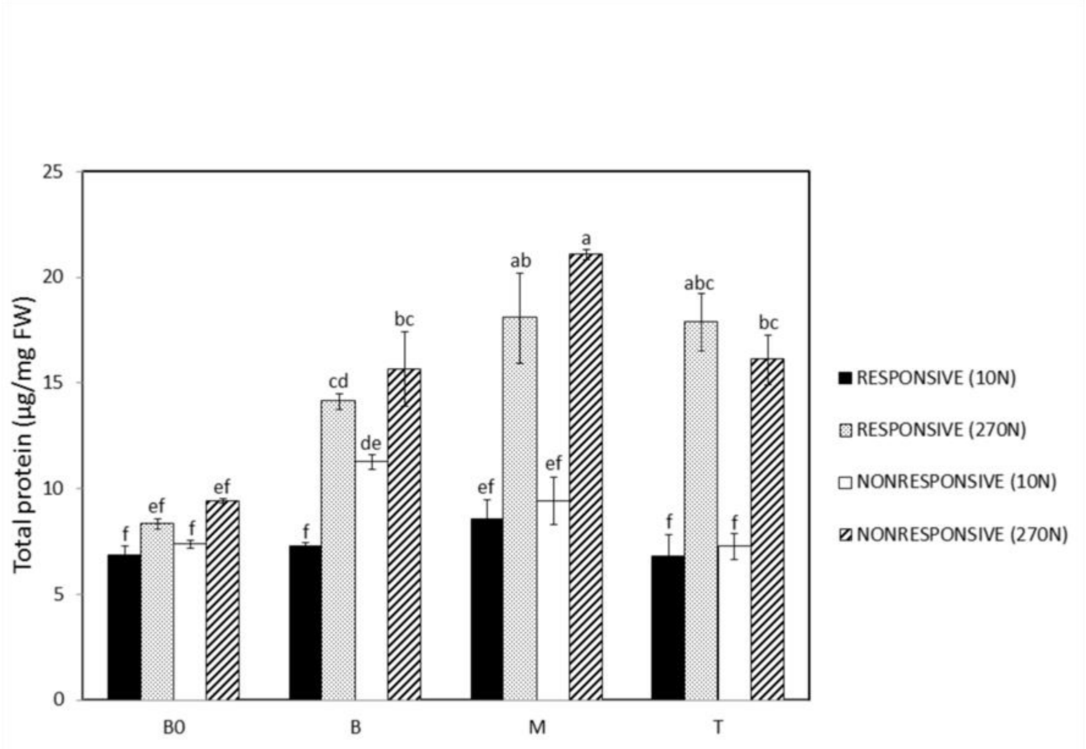

Supplementary Figure S7. Protein content along sugarcane leaf blade. 10N and 270N correspond to 10 and 270 mg of N per kg of sand treatments, respectively. B0, Base "zero"; B, Base; M, Middle; T, Tip. FW, fresh weight. Data are presented as the mean ± SE. Letters indicate statistical significance using ANOVA followed by Fisher test ($n = 3$; $p \leq 0.05$).

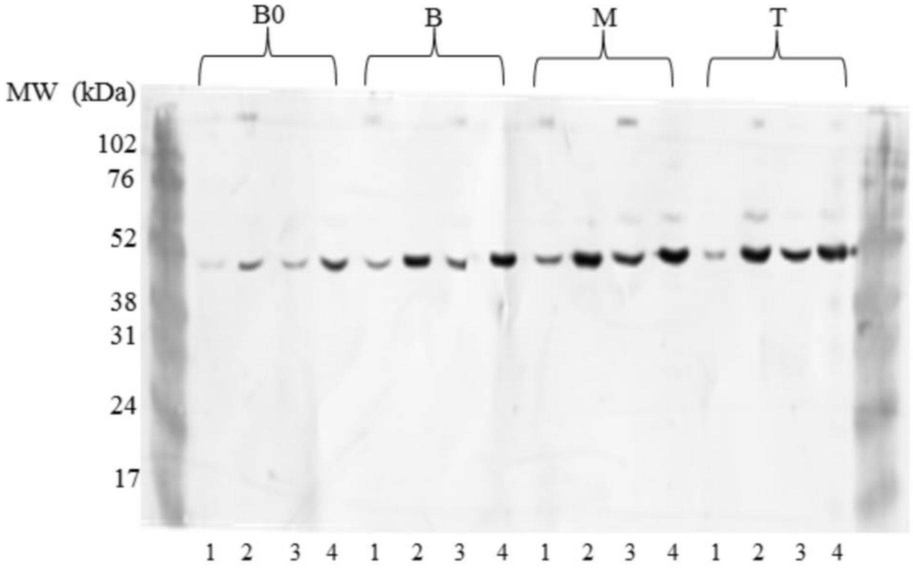

Supplementary Figure S8: Western blot membrane of ribulose-1,5-bisphosphate carboxylase/oxygenase (RubisCO). The commercial peptide sequences of RubisCO large subunit form I (Agrisera, SWE) were used. B0, Base "zero"; B, Base; M, Middle; T, Tip. 1, Responsive with 10 mg of N; 2, Responsive with 270 mg of N; 3, Nonresponsive with 10 mg of N; 4, Nonresponsive with 270 mg of N. MW, Molecular weight.

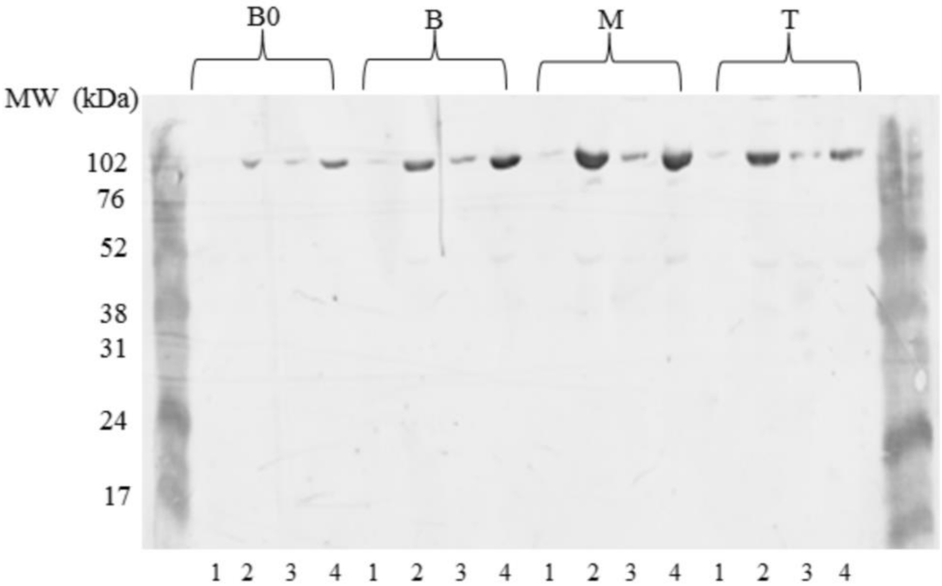

Supplementary Figure S9: Western blot membrane of phosphoenolpyruvate carboxylase (PEPcase). The commercial peptide sequences of PEPC1 (Agrisera, SWE) were used. B0, Base "zero"; B, Base; M, Middle; T, Tip. 1, Responsive with 10 mg of N; 2, Responsive with 270 mg of N; 3, Nonresponsive with 10 mg of N; 4, Nonresponsive with 270 mg of N. MW, Molecular weight.

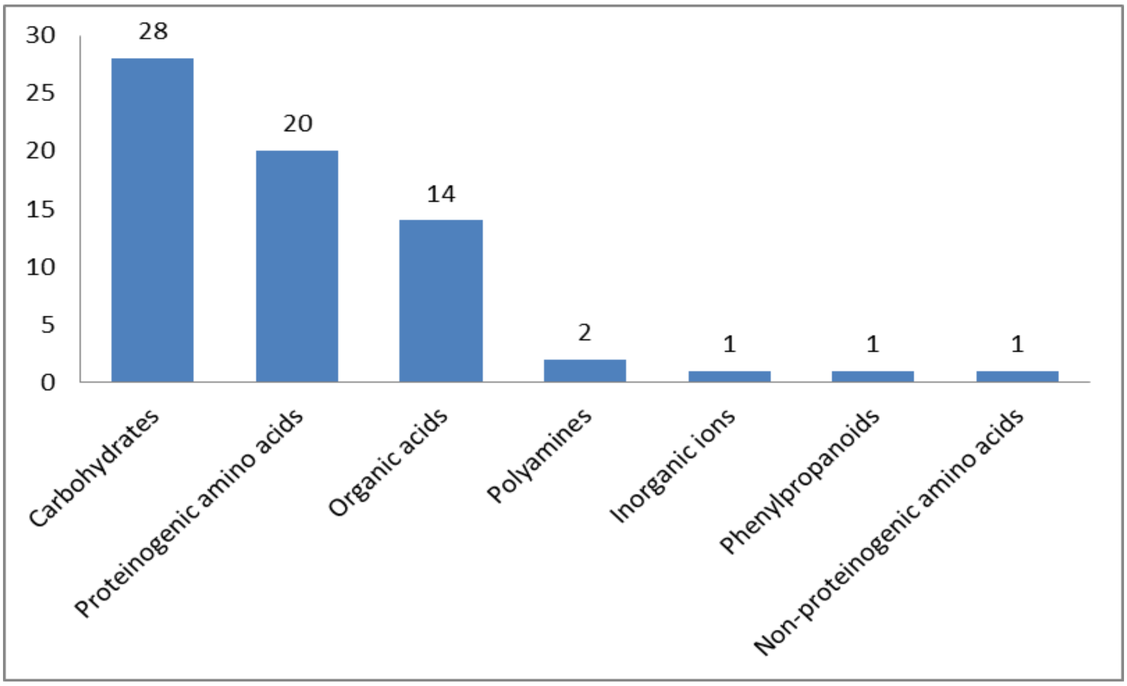

Supplementary Figure S10. Distribution and chemical classification of the metabolites identified by gas chromatography mass spectrometry (GC-MS).

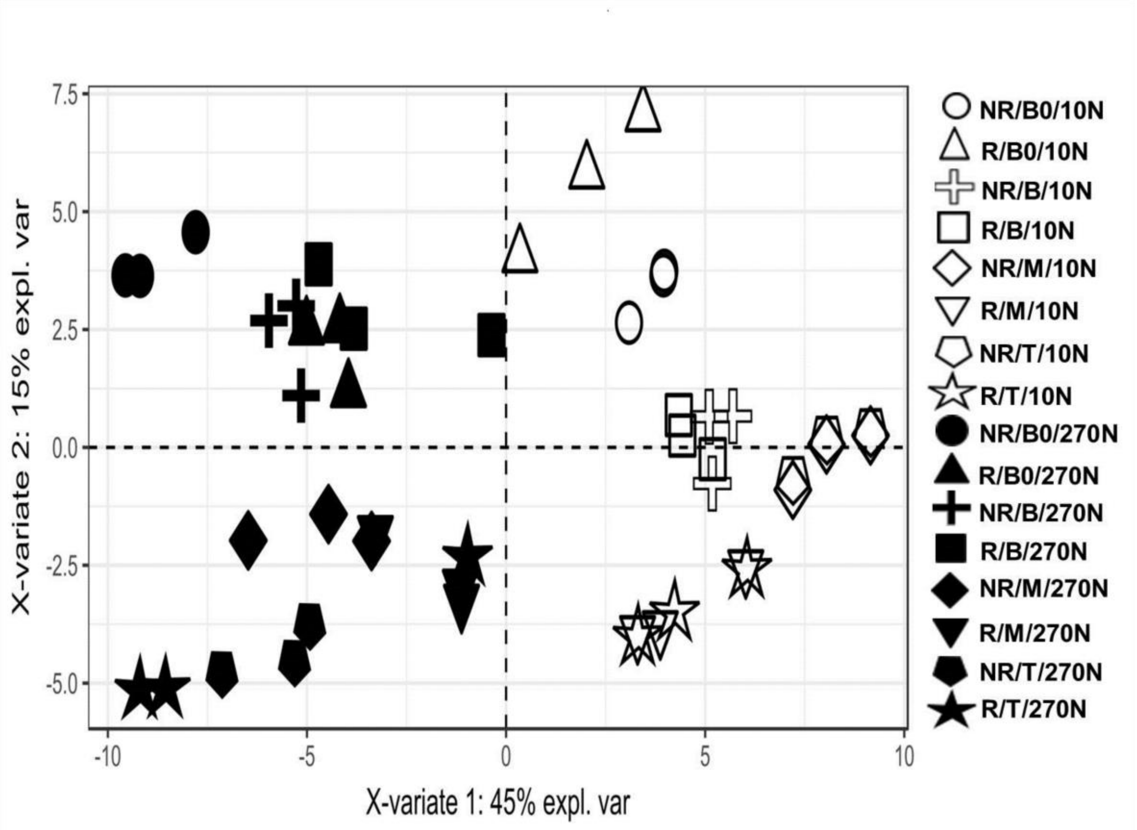

Supplementary Figure S11. Results of partial least square discriminant analysis (PLS-DA) in relation to metabolites detected by GC-MS. R, responsive. NR, nonresponsive. B0, Base “zero”; B, Base; M, Middle; T, Tip. 10N and 270N correspond to 10 and 270 mg of N per kg of sand treatments, respectively.

Supplementary Table S2. Result from over representation analysis (ORA). The fold enrichment was calculated among all metabolites detected (67) and the metabolites present in each pathway of the customized library. Total; metabolite number of each reference pathway. Hits; metabolite number in relation to reference pathway.

Metabolic pathway	Total	Expected	Hits	Raw p	Holm p	FDR
Protein biosynthesis	20	1.24	17	3.58E-19	1.43E-17	1.43E-17
ABC transporters	96	5.96	30	2.35E-16	9.18E-15	4.71E-15
Biosynthesis of plant secondary metabolites	127	7.89	32	1.83E-14	6.97E-13	2.45E-13
Proteinogenic amino acids degradation	94	5.84	24	9.76E-11	3.61E-09	9.76E-10
Sugar metabolism	41	2.55	16	2.44E-10	8.77E-09	1.95E-09
Urea cycle	19	1.18	9	4.49E-07	1.57E-05	3.00E-06
Plant hormones biosynthesis	71	4.41	16	1.83E-06	6.22E-05	9.51E-06
Phenylpropanoids biosynthesis	55	3.41	14	1.90E-06	6.27E-05	9.51E-06
Carbon metabolism	95	5.9	18	5.49E-06	0.000176	2.44E-05
2-oxocarboxylic acid metabolism	97	6.02	17	3.26E-05	0.00101	0.00013
TCA cycle	19	1.18	7	7.21E-05	0.00216	0.000262
Fructose and mannose metabolism	54	3.35	11	0.000261	0.00758	0.000871
Photosynthesis	32	1.99	8	0.00046	0.0129	0.00142
Amino acids metabolism	49	3.04	10	0.000508	0.0137	0.00145
Starch and sucrose metabolism	51	3.17	10	0.000715	0.0186	0.00191
Photorespiration	16	0.993	5	0.00203	0.0508	0.00508
Glucose-alanine cycle	11	0.683	4	0.00322	0.0772	0.00757
Malate-aspartate shuttle	8	0.497	3	0.0102	0.235	0.0227
Glutathione metabolism	45	2.79	7	0.0177	0.39	0.0373
Phenylalanine and tyrosine metabolism	13	0.807	3	0.0418	0.877	0.0835
Ascorbate and alderate metabolism	24	1.49	4	0.0562	1	0.102
Gluconeogenesis	24	1.49	4	0.0562	1	0.102
Amino sugar and nucleotide sugars metabolism	82	5.09	8	0.128	1	0.222
Mitochondrial electron transport chain	14	0.869	2	0.214	1	0.357
Glycolysis	18	1.12	2	0.309	1	0.494
Propanoate metabolism	48	2.98	4	0.348	1	0.535
Valine leucine and isoleucine degradation	35	2.17	3	0.372	1	0.551
Nucleotide sugars metabolism	9	0.559	1	0.44	1	0.628
Aromatic compounds degradation	31	1.92	2	0.585	1	0.807
Pentose phosphate pathway	17	1.06	1	0.667	1	0.832
Porphyrin and chlorophyll metabolism	90	5.59	5	0.674	1	0.832
Tyrosine metabolism	38	2.36	2	0.698	1	0.832
Nitrogen metabolism	19	1.18	1	0.707	1	0.832
Pyruvate metabolism	19	1.18	1	0.707	1	0.832
Glycerolipid metabolism	42	2.61	2	0.75	1	0.857
Tryptophan metabolism	34	2.11	1	0.891	1	0.99
Carotenoid biosynthesis	73	4.53	1	0.992	1	1
Plastoquinone biosynthesis	74	4.59	1	0.993	1	1
Fatty acid metabolism	105	6.52	2	0.993	1	1

Green cells represent the significant pathway at P < 0.05 level and FDR ≤ 0.1; FDR; False discovery rate.

References

1. Lê Cao, K. A., González, I. & Déjean, S. IntegrOmics: An R package to unravel relationships between two omics datasets. Bioinformatics 25, 2855–2856 (2009).

2. Farquhar, G. D. On the Nature of Carbon Isotope Discrimination in C 4Species. Aust. J. Plant Physiol. 10, 205–226 (1983).

3. Robinson, N. et al. Sugarcane genotypes differ in internal nitrogen use efficiency. Functional Plant Biol. 34(12):1122–1129 , (2007).
